# Characteristics of Primary Cutaneous T-Cell Lymphoma in Iran: A 10-Year Retrospective Study

**DOI:** 10.1155/2014/820921

**Published:** 2014-12-24

**Authors:** Farahnaz Fatemi Naeini, Hamidreza Sadeghiyan, Mohsen Pourazizi, Jamshid Najafian, Bahareh Abtahi-Naeini

**Affiliations:** ^1^Skin Diseases and Leishmaniasis Research Center, Department of Dermatology, Faculty of Medicine, Isfahan University of Medical Sciences, Isfahan, Iran; ^2^Students' Research Committee, Isfahan University of Medical Sciences, Isfahan, Iran; ^3^Students' Research Committee, Semnan University of Medical Sciences, Semnan, Iran; ^4^Cardiovascular Research Center, Cardiovascular Research Institute, Isfahan University of Medical Sciences, Isfahan, Iran

## Abstract

*Background*. Primary cutaneous T-cell lymphomas (CTCLs) are a group of extranodal non-Hodgkin lymphomas that may be present in the skin without any evidence of extracutaneous disease. The aim of this study was to evaluate the epidemiological characteristics of primary CTCL in Isfahan, Iran.* Method*. A total of 95 patients who were diagnosed as having primary CTCL were recruited during a 10-year period (2003–2013) and were classified according to the new WHO-EORTC criteria.* Results*. The patient group consisted of 43 (44.8%) males and 53 (55.2%) females, which indicated a female predominance (M : F ratio 1 : 1.2). The mean age at the time of diagnosis was 41.78 ± 16.88 years (range: 7–84 years). Prior to diagnosis, the lesions had persisted for a mean of 8.34 ± 4.38 years (range: 0–55 years). The age at peak diagnosis was 20–40 years (43%). The most frequent subtypes were mycosis fungoides (MF) (88.5%). Four patients died from CTCL-related complications.* Conclusions*. The distinguishing epidemiologic characteristics of primary CTCL, particularly those MF, in Iran, are the absence of a male predominance and lower age at diagnosis. This is likely because of the characteristic ethnic group diversity and increased susceptibility among younger population.

## 1. Introduction

Primary cutaneous T-cell lymphomas (CTCLs) are a heterogeneous group of lymphomas that may be present in the skin without any evidence of extracutaneous disease at the time of diagnosis [1, 2]. Skin is the second most common site for extranodal non-Hodgkin lymphoma (NHL) [1]. In contrast to nodal NHL, most of which are B-cell-derived, approximately 75% of the primary cutaneous lymphomas (PCL) are T-cell-derived, two-thirds of which are mycosis fungoides (MF) and Sézary syndrome (SS) [3, 4].

The annual incidence of MF in the United States is 4.1 per 1,000,000 person-years [4]. The incidence rate in Europe is somewhat less [5]. Between the years 2007 and 2008, the incidence rate of MF in Isfahan was 3.91 per 1,000,000 individuals [6]. Currently, the World Health Organization-European Organization for the Research and Treatment of Cancer (WHO-EORTC) classification guidelines are used for the classification of cutaneous lymphomas [3, 7].

Reports of large-scale epidemiologic studies of the various subtypes of primary CTCLs and their relative frequencies have typically described population in developed countries. Data from developing countries are particularly scarce [4, 8], and studies of the epidemiological characteristics of primary CTCL in Iran are limited to one report that described a group of patients in Tehran [9]. Thus, the present study, which was conducted at the Cutaneous Lymphoma Center of Isfahan University of Medical Sciences, was the first to investigate the clinical epidemiological characteristics of primary CTCL in Iran.

## 2. Materials and Methods

In this single-center study, which was conducted from 2003 to 2013, 118 patients with cutaneous lymphoma were analyzed in the Academic Referral Center for Cutaneous Lymphoma of the Al-Zahra Hospital of the Isfahan University of Medical Sciences and were classified according to the clinical, histopathological, immunophenotyping, and molecular criteria of the WHO-EORTC [3, 10].

Patients with cutaneous B-cell lymphomas, secondary cutaneous lymphomas, clonal dermatitis, and cutaneous lymphoid hyperplasia and cases with poor material or inadequate information were excluded from this study. The remaining 95 cases were analyzed.

The clinical data analyzed included age at the time of diagnosis, age at onset of the cutaneous lesion, sex, and status of the disease at the last follow-up examination.

Hematoxylin and eosin stained slides and immunohistochemical staining performed for B-cell markers, T-cell markers (CD20 and CD3 and/or CD45RO), CD4, CD8, CD30, Ki 67, CD5, and CD1a were evaluated by an expert dermatopathologist. Polymerase chain reaction analyses of T-cell receptor (TCR) gene rearrangement were reviewed. In all patients, the presence of extracutaneous disease at the time of diagnosis had been excluded by standard staging procedures. Stage workup (chest radiology, bone marrow biopsy, and computed tomography scan of chest, abdomen, and pelvis) data were obtained by reviewing clinical records. To determine the stage of PCTCL, tumor-node-metastasis (TNM) system was used as proposed by Olsen et al. [11].

Results were reported as mean ± standard deviation (SD) for the quantitative variables and percentages for the categorical variables. The groups were compared using the *t*-test for the continuous variables and the chi-square test (or Fisher's exact test if required) for the categorical variables. *P* values of 0.05 or less were considered statistically significant. All the statistical analyses were performed using SPSS version 16.0 (SPSS Inc., Chicago, IL, USA) for Windows.

## 3. Results

Between the years 2003 and 2013, a total of 95 cases of primary CTCLs were diagnosed in the Cutaneous Lymphoma Center of the Dermatology Department of the Isfahan University of Medical Sciences, Iran.  Table 1 summarizes the demographic profiles and basic data of the 95 patients included in our study. Of these 95 patients, 53 (55.8%) were females. Thus, a female predominance was noted (M : F ratio 1 : 1.2). The mean age at the time of diagnosis was 41.7 ± 16.9 years (range: 7–84 years) and the lesion had persisted for a mean of 4.4 ± 8.3 years (range: 0 to 55 years) before the diagnosis was made (Table 1).

**Figure 1:**
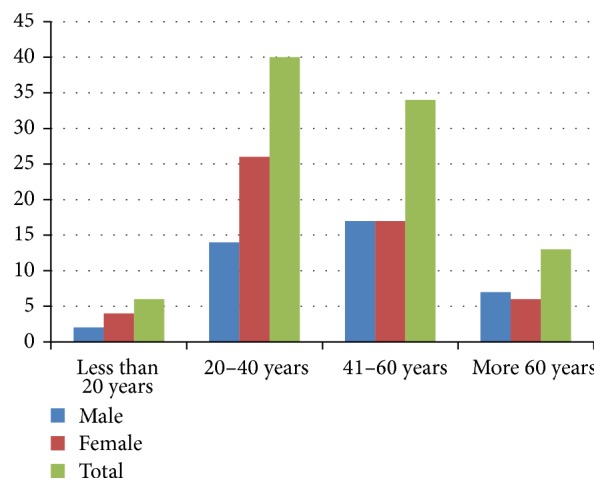
The age distribution of patients with primary CTCL.

The mean latent period in men (2.3 ± 4.1 years) was significantly different from that in women (5.9 ± 10.1 years) (*P* = 0.02). Of the 95 patients, 43.5% were in the age group 20 to 40 years and 35.9% were in the age group 41 to 60 years. The age group 20 to 40 years was more predominant among females. The age at the peak of diagnosis was between 20 and 40 years. Only one patient was older than 80 years (Figure 1).

**Figure 2:**
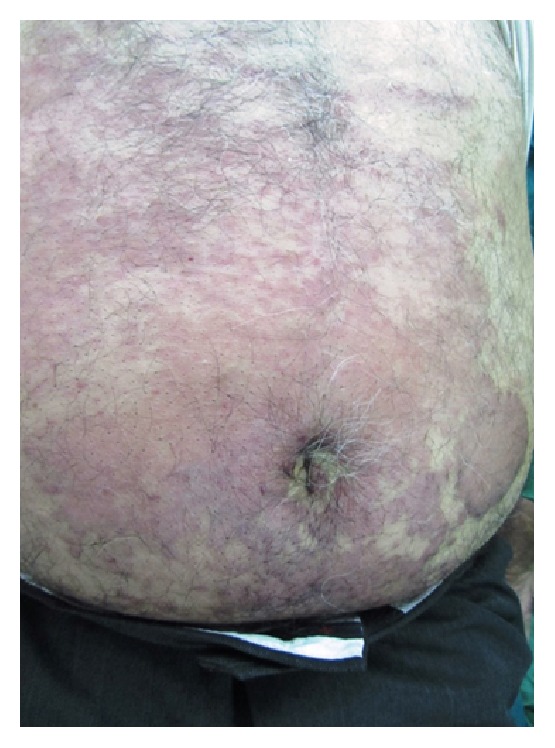
Mycosis fungoides. Erythematous plaques affected the trunk area.

The frequency of each subtype of primary CTCL (new WHO-EORTC classification) is shown in Table 2. Of the 95 cases of primary CTCL, 85 patients (89.5%) developed MF (Table 2). At the time of diagnosis, the most prevalent skin lesions were erythematous scaly patch (in 49% of the patients) and erythematous plaques (in 23% of the patients). The lesions preferentially affected the trunk area (Figure 2).

**Figure 3:**
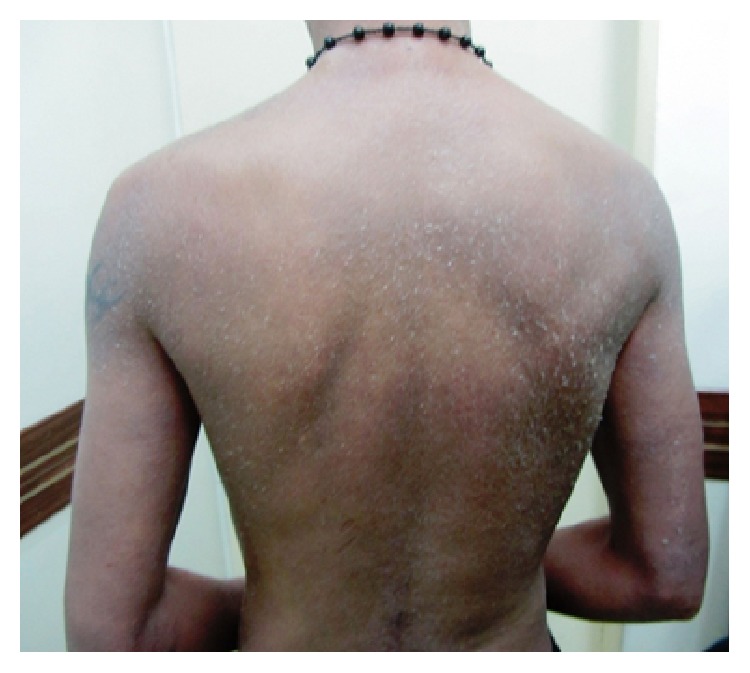
Erythrodermic mycosis fungoides. Advanced stage of mycosis fungoides in a 25-year-old man.

According to the TNM classification, among the 85 cases of MF, 82 cases (87.2%) were early stage (stage I + IIA) and the remaining cases (12.8%) were advanced stage (stage IIB + III + IV) (Figure 3). There was no significant correlation between patient's sex and the disease stage (*P* > 0.05).

Approximately 10% of the patients had a high level of lactate dehydrogenase. Of the 95 patients, 6 cases had acquired ichthyoses. Lymphadenopathy (7 cases), jaundice (5 cases), and hepatosplenomegaly (1 case) were the extracutaneous manifestation of the disease in our patients.

TCR gene rearrangement clonality was seen in 9 out of 25 cases. All patients were treated with one or more of the modalities, including topical corticosteroid therapy, topical nitrogen mustard, topical BCNU, radiotherapy, NB ultraviolet (UV) radiation therapy, psoralen plus UVA (PUVA) therapy, retinoid-PUVA therapy, interferon-PUVA therapy, interferon alpha-2a, and systemic mono-/polychemotherapy. Among the treatments, topical carmustine (38.5%), NB-UVB (36.5%), and PUVA (26%) were the more commonly used modalities. Four patients died from lymphoma. Primary CTCL-related deaths were most commonly associated with SS and erythrodermic MF.

## 4. Discussion

The sex and age preference of primary CTCLs observed in this study of Iranian population were different from those reported earlier. There was a higher incidence of primary CTCLs and MF in the fourth to sixth decades. Earlier studies have found that the peak age at presentation of the disease was 55–60 years [12, 13]. Although MF usually affects older adults with a median age of more than 50 years, median ages of 33 (Singapore) [14] and 35.2 (Kuwait) [15] years have also been reported. In our series, approximately 40% of the patients were diagnosed at 20–40 years of age. Thus, in the present study, the mean age at diagnosis was younger than that reported for the Western population (between the 4th and 6th decades) [16, 17] and Turkey (45.6 years) [18]. These results suggested that the age at presentation of the disease was lower in Asian countries than in Western countries.

Nearly all studies of primary CTCLs and MF have found a male predominance, with the reported male to female ratio ranging from 1.3 : 1 to 2 : 1 [2, 19, 20]. In contrast, the male to female ratio in the group of patients we studied was 1 : 1.2, which is similar to the ratio found in a previous study (1 : 1.33) of the incidence rate of MF in Isfahan [6]. This difference might be due to the characteristic ethnic group diversity and host susceptibility. However, further studies with larger sample size are needed to identify the factors that contributed to this difference.

MF/SS was the most common type of primary CTCL in our patients, with other types occurring less frequently. This predominance of MF might be due to the interplay of a different set of environmental and genetic factors in Iran. The rates of occurrence of various subtypes of cutaneous lymphoma in Asia are considered to be significantly different from those in the Western countries [21]. The results of the present study on Iranian patients are substantially different from those obtained for Asian and Western populations. Compared with our patient group, higher rates of cutaneous NK/T-cell lymphomas such as extranodal NK/T-cell lymphoma and subcutaneous panniculitis-like T-cell lymphoma (SPTCL) have been found in Korean and Japanese populations [22, 23]. Notably, similar to that found in several European studies, the incidence rate of SPTCL in our study was nearly zero [5, 9].

A significantly high proportion of our patients complained about the onset of their disease before 20 years of age. This is similar to the results of an earlier study of patients in Kuwait [15]. The possibility that environmental pollution may have contributed to the increased incidence rates in younger population cannot be ruled out.

Primary CTCL is commonly misdiagnosed as benign lesions such as eczema and psoriasis. Therefore, if not evaluated and biopsied by a specialist with a high index of suspicion, some patients may never be diagnosed correctly. This is consistent with a previous study that found a significant correlation between primary CTCL incidence and physician density and a strong correlation between primary CTCL incidence and medical specialist density [24]. Primary CTCL also appears to have been underdiagnosed in areas with fewer dermatologists [25]. Therefore, follow-up studies are needed to quantify the contributions of residential and occupational exposures to primary CTCL.

## 5. Conclusions

The distinguishing epidemiologic characteristics of primary CTCL, especially MF, in Iran are the lack of male predominance and lower age. This is likely because of the ethnic group diversity and the characteristic susceptibility of younger population to primary CTCL. Further analyses of the environmental and genetic factors that contribute to the development of various forms of primary CTCL in Iranian population are critical for the prevention and treatment of primary CTCL in Iran and in other parts of the world.

## Figures and Tables

**Table 1:** Demographic profile and basic data of 95 patients with primary CTCL.

Age (years)	
Range	7–84
Median	40.5
Mean (±SD)	41.7 ± 16.9
Sex	
Male	42 (44.2%)
Female	53 (55.8%)
Latent period (years)	
Range	0–55
Median	1
Mean (±SD)	4.43 ± 8.38
TNM stage	
IA	38 (40%)
IB	43 (45.7%)
IIA	2 (2.1%)
IIB	3 (3.2%)
III	4 (4.2%)
IV	5 (5.3%)

**Table 2:** Characteristics of PCTCL by WHO-EORTC classification in Isfahan, Iran.

Classification	Number of cases (%)	M	F	Age, year mean (±SD)	Latent period, year mean (±SD)
Mycosis fungoides	85 (89.5)	39	46	41.4 (16.9)	4.7 (8.7)
Folliculotropic mycosis fungoides	1 (1.1)	0	1	45	1
Pagetoid reticulosis	0	0	0	0	0
Granulomatous slack skin	0	0	0	0	0
Sézary syndrome	4 (4.2)	2	2	61 (16.7)	1
Lymphomatoid papulosis	1 (1.1)	0	1	30	4
Primary cutaneous anaplastic large cell lymphoma	2 (2.1)	0	2	31.5 (2.1)	4 (5.6)
Peripheral T-cell lymphoma	1 (1.1)	1	0	30	0
Primary cutaneous CD4+ small/medium pleomorphic T-cell lymphoma	0	0	0	0	0
Primary cutaneous NK/T-cell lymphoma, nasal type	1 (1.1)	0	1	32	0
Subcutaneous panniculitis-like T-cell lymphoma	0	0	0	0	0
Primary cutaneous γ/δ T-cell lymphoma	0	0	0	0	0

Total	95 (100)	42	53	41.7 (16.9)	4.4 (8.3)
